# Insomnia, Excessive Sleepiness, Excessive Fatigue, Anxiety, Depression and Shift Work Disorder in Nurses Having Less than 11 Hours in-Between Shifts

**DOI:** 10.1371/journal.pone.0070882

**Published:** 2013-08-15

**Authors:** Maria Fagerbakke Eldevik, Elisabeth Flo, Bente Elisabeth Moen, Ståle Pallesen, Bjørn Bjorvatn

**Affiliations:** 1 Norwegian Competence Center for Sleep Disorders, Haukeland University Hospital, Bergen, Norway; 2 Department of Public Health and Primary Health Care, University of Bergen, Bergen, Norway; 3 Department of Psychosocial Science, University of Bergen, Bergen, Norway; 4 Department of Occupational Medicine, Haukeland University Hospital, Bergen, Norway; University of Alabama at Birmingham, United States of America

## Abstract

**Study objective:**

To assess if less than 11 hours off work between work shifts (quick returns) was related to insomnia, sleepiness, fatigue, anxiety, depression and shift work disorder among nurses.

**Methods:**

A questionnaire including established instruments measuring insomnia (Bergen Insomnia Scale), sleepiness (Epworth Sleepiness Scale), fatigue (Fatigue Questionnaire), anxiety/depression (Hospital Anxiety and Depression Scale) and shift work disorder was administered. Among the 1990 Norwegian nurses who participated in the study; 264 nurses had no quick returns, 724 had 1–30 quick returns and 892 had more than 30 quick returns during the past year. 110 nurses did not report the number of quick returns during the past year. The prevalence of insomnia, excessive sleepiness, excessive fatigue, anxiety, depression and shift work disorder was calculated within the three groups of nurses. Crude and adjusted logistic regression analyses were performed to assess the relation between quick returns and such complaints.

**Results:**

We found a significant positive association between quick returns and insomnia, excessive sleepiness, excessive fatigue and shift work disorder. Anxiety and depression were not related to working quick returns.

**Conclusions:**

There is a health hazard associated with quick returns. Further research should aim to investigate if workplace strategies aimed at reducing the number of quick returns may reduce complaints among workers.

## Introduction

In Europe and the US today, shift work and irregular working hours have become the rule rather than the exception. In fact, only about 25% of the workforce in Europe has regular day work [1], [2]. Many employees have flexible working hours, while others are shift workers or workers with irregular working hours. In Norway, 32.7% of all employees worked shifts or irregular hours in 2011 [3]. When so many employees are working shifts, it is important to study and assess the consequences shift work may have on health. Several studies have attested to the negative health effects of shift work on both physical and mental problems. Recent reviews suggest that shift work is related to cardiovascular diseases [4]. Shift work has further been associated with cancer, in particular breast cancer [5], [6]. Furthermore, studies have shown associations between shift work and gastrointestinal complaints, peptic ulcer disease, diabetes type 2 and rheumatoid arthritis [7], [8], [9]. Regarding mental health and sleep problems, there is strong evidence that shift work negatively influences sleep, alertness and fatigue [10]. A nonspecific association with mental health is also found [11]. In a recent study, we found that shift work disorder (SWD) was significantly associated with anxiety, depression and quick returns [12]. Another study has shown an improvement in mental workability in relation to a reduction in number of quick returns [13]. The physical and mental health effects of shift work may be caused by circadian disturbance and/or sleep deprivation related to irregular working hours. Shift work may disturb health by several psychosocial, behavioral and psychological mechanisms [1].

Since sleep and rest are important factors for good health, short time for recovery between each shift may have a negative impact on health. Short rest periods in-between shifts are denoted differently in the literature [14]. We have chosen to use the term *quick returns* in the present study. While we do not know the exact prevalence of workers affected by quick returns, one study suggests that quick returns are common within certain occupations [15]. Typically, quick returns are denoted as a short time span between two consecutive shifts worked by the same employee. Quick returns often occur as part of a rotational schedule, typically when an evening shift is followed by a day shift or a night shift is followed by an evening shift. Also, extended work hours with late end times may result in few hours of rest before starting on the next shift. Strategic planning of the work schedules may keep quick returns at a minimum [13]. Eleven hours of rest separating work shifts are the recommended minimum of time by the European Union's working time directive [16]. In Norway, this is expressed indirectly through the Work and Environment Act, telling that no workers should be at work for more than 9 hours during a 24-hour period. In the present study, quick returns were defined as less than eleven hours in-between shifts.

There is ample evidence attesting to the relationship between performance, accidents and sleep. Some studies suggest that quick returns cause insufficient sleep among the workers [17]. This might cause reduced work performance, and quick returns may represent a risk of errors and accidents. A review by Kecklund et al. indicates that quick returns reduce sleep duration. It is suggested that 16 hours of free time is needed in order to have 7–8 hours of sleep [18].

Quick returns are often associated with rotation between shifts, which has been shown to be detrimental for the health of shift workers [10]. A rotation between different shifts disrupts the endogenous circadian rhythm [19]. Re-adaptation from night- to daytime activity may take several days [20]. Quick returns may encumber this entrainment, and may thus contribute to the sleep and sleepiness problems observed in shift workers [21]. Therefore, awareness of how quick returns can affect sleep and health is essential when it comes to work scheduling. Since few studies have specifically explored how quick returns in particular affect sleep and mental health, there is a need of further research on this topic.

The aim of the present study was to assess whether insomnia, excessive sleepiness, excessive fatigue, anxiety, depression and shift work disorder were associated with quick returns among shift workers. We hypothesized that increased number of quick returns would be associated with an increased prevalence of such complaints.

## Methods

### Procedure and participants

A sample of 5400 nurses from the Norwegian Nurses Organisation's membership register was used. All selected nurses worked at least 50% of full time equivalent position, equalling 17 h 45 min per week for nurses working shift-rotations and 18 h and 45 min per week for those working permanent day shifts. Nurses were randomly selected from five equal strata based on number of years since the completion of basic nursing education (0–1 y, 1.1–3 y, 3.1–6 y, 6.1–9 y, and 9.1–12 y). The nurses were working in different hospitals throughout Norway: half of the nurses came from Western Norway, the other half from the rest of the country. The questionnaire, an information letter and a prepaid return envelope were sent by mail to these nurses during the winter 2008/2009. All nurses were informed that participants would be part of a lottery where 50 individuals would win 500 NOK (about $ 85). As the questionnaire was available on the Internet, the nurses had the opportunity to respond both online and by postal mail. Two reminders were sent to those who did not respond. A total of 2059 nurses responded, which gave a response rate of 38.1%. 69 of the nurses who responded had to be excluded from the study, either because they didn't report their fraction of full position or because they were working less than 50%. As a consequence, all the analyses in this study were based on a sample of 1990 nurses. However, only the nurses who provided information on how many shifts separated by less than 11 hours they had worked the past year were included in the specific analyses. Demographic data were registered, including age, gender, number of night shifts during the past year, fraction of full position and number of quick returns during the past year (Table 1). The questionnaire included a large number of questions, since this study was part of a larger project. Only the questions and instruments relevant for the present study are reported here.

**Table 1:** Distribution of gender, fraction of full position, nurses working quick returns, age, number of working nights and number of quick returns among nurses (n = 1990).

	N	Valid percent
Gender		
*Male*	190	9.6%
*Female*	1792	90.4%
Fraction of full position		
*50–75%*	577	29.0%
*76–90%*	269	13.5%
*>90%*	1144	57.5%
Number of nurses working quick returns	1616	81.2%
	**Mean (Range)**	**SD**
Age	33.1 (21–63)	8.2
Number of working nights past year	28.7 (0–206)	29.3
Number of quick returns past year	33.2 (0–156)	27.8

N =  Number.

SD =  Standard deviation.

### Instruments

#### Bergen Insomnia Scale (BIS)

BIS comprises six questions; each having an 8-point scale where the number between 0 and 7 indicates days per week a certain symptom has been experienced [22]. The total score ranges from 0 to 42. A score of 3 or higher on at least one of the first 4 questions (night-time problems), combined with the score of 3 or higher on at least one of the 2 last questions (daytime consequences) is regarded as fulfillment of the insomnia criteria. The items are based on the American Psychiatric Association (APA)'s Diagnostic and Statistical Manual of Mental Disorders-IV-TR inclusion criteria for insomnia [23]. The Cronbach's alpha for the BIS was .83 in the present study.

#### Epworth Sleepiness Scale (ESS)

ESS is a scale that describes the likelihood of falling asleep or dozing off in eight situations [24]. The participants indicate this likelihood by ticking off a number from 0 to 3, where 0 represents no risk of dozing off, and where 3 represents a high risk of dozing off. A total score of 11 or higher is suggestive of excessive sleepiness. The ESS has in several studies shown high reliability and validity. A validated Norwegian version was used [25]. The Cronbach's alpha for the ESS was .73 in the present study.

#### Fatigue Questionnaire (FQ)

The FQ consists of 13 items [26]. The first 11 items measure physical and mental fatigue, using a Likert scale ranging from 0 to 3. The items were dichotomized (0,0,1,1) so that “0” and “1” were recoded into “0” and “2” and “3” were recoded into “1”, respectively. The two last items measure for how long and how often the feeling of fatigue has been present. A sum score exceeding 3 (dichotomized score) on the 11 first items is considered as excessive fatigue. The FQ is commonly used as a tool to measure fatigue [26]. A Norwegian version was used [27]. In the present study, the Cronbach's alpha was .89 for the FQ.

#### Hospital Anxiety and Depression Scale (HADS)

HADS consists of 14 items, of which 7 measure anxiety and 7 measure depression. The response alternatives range from 0 to 3, indicating the degree a specific symptom has been experienced during the last week [28]. A score of 8 or higher on the HADS subscales for anxiety and depression respectively is considered as a clinical cutoff. A validated Norwegian version of the HADS was used [29]. In the present study, the Cronbach's alpha scores for the anxiety and depression subscales were .82 and .81, respectively.

#### Shift work disorder (SWD)

Three questions were used to differentiate between participants with SWD and non-SWD participants. The questions used are developed for the particular purpose of assessing SWD in epidemiologic studies [12], [30], and they adhere to criteria for SWD found in the International Classification of Sleep Disorders, second edition [31]. To fulfill the SWD criteria, the participants had to answer “yes” to all of the following three questions: (1) Do you experience difficulties with sleeping or excessive sleepiness? (2) Is the sleep or sleepiness problem related to a work schedule where you have to work when you would normally sleep? (3) Has this sleep or sleepiness problem related to your work schedule persisted for at least one month? All three questions had a “yes/no” answer format.

### Statistical Analyses

SPSS version 20 was used for the statistical analyses. The number of quick returns during the past year was used to divide the nurses into three groups: 0 shifts, 1–30 shifts, 31 or more shifts. These groups were selected based on the median of quick returns during the past year (30.0). 2×2 chi-square analyses (continuity correction) were calculated comparing the three groups of nurses (0 compared to 1–30, 0 compared to >31 and 1–30 compared to >31 quick returns) in relation to insomnia, excessive sleepiness, excessive fatigue, anxiety, depression and SWD. Also, to compare the same three groups of nurses, we calculated the mean total score and standard deviations for the summed scores for the BIS, the ESS, the FQ, the HADS-A and the HADS-D. Effect sizes (Cohen's d) for the group comparisons were calculated for categorical variables (based on the chi-square statistics and number of participants) and continuous variables (based on the means and standard deviations).

We conducted separate logistic regression analyses with insomnia, excessive sleepiness, excessive fatigue, anxiety, depression and SWD as the dependent variable. As predictor variables, we included age, gender, number of night shifts the past year, years with night work, fraction of full position and number of quick returns. First, all the variables were entered separately in crude analyses. Secondly, all the variables were entered together in an adjusted analysis. Additionally, we conducted adjusted analyses with quick returns as a continuous variable. The odds ratio was considered statistically significant if the 95% confidence interval did not include 1.00.

### Ethical statement

The Regional Committee for Medical and Health Research Ethics, Health Region West (REK-Vest) approved this study. Informed consent was obtained in written form.

## Results

Table 1 shows an overview of the study population. A total of 81.2% of the nurses in the present study reported quick returns. The mean number of quick returns past year was 33.2.

Insomnia, excessive sleepiness, excessive fatigue and shift work disorder were more prevalent among nurses with a high number of quick returns (Table 2). Similarly, there were increases in the mean total scores of the BIS, the ESS and the FQ with increased number of quick returns, but only the ESS showed adequate effect sizes (Table 2). The prevalence and total scores for anxiety and depression did not show major differences within the three groups.

**Table 2:** Prevalence, mean total score, standard deviation (SD) and effect sizes of insomnia, sleepiness, fatigue, anxiety, depression and shift work disorder across three groups of nurses working different numbers of quick returns.

*Number of quick returns*	0 (N = 290)	1–30 (N = 724)	>30 (N = 892)	Effect sizes (Cohen's d)		
				1–30 vs 0	>30 vs 0	>30 vs 1–30
Insomnia	45.0%	53.5%	57.8%	0.17	0.26	0.08
BIS total score (SD)	13.2 (9.9)	13.3 (8.1)	13.8 (7.9)	0.02	0.07	0.06
Excessive Sleepiness	18.8%	28.1%	30.9%	0.20	0.25	0.06
ESS total score (SD)	7.2 (3.5)	8.4 (3.7)	8.9 (3.6)	0.33	0.47	0.14
Excessive fatigue	35.5%	37.2%	43.4%	0.02	0.14	0.12
Fatigue total score (SD)	13.5 (4.5)	13.3 (4.1)	13.9 (4.4)	−0.05	0.09	0.14
Anxiety	19.2%	18.0%	20.7%	−0.04	0.01	0.07
HAD-A total score (SD)	4.5 (3.6)	4.6 (3.3)	4.8 (3.6)	0.03	0.08	0.06
Depression	8.4%	8.4%	9.0%	0.00	0.02	0.02
HAD-D total score (SD)	2.8 (3.1)	2.7 (2.9)	2.8 (2.9)	−0.03	0.00	0.03
SWD	24.5%	35.4%	45.1%	0.24	0.43	0.20

N =  Number.

SD =  Standard deviation.

BIS =  Bergen Insomnia Scale.

ESS =  Epworth sleepiness Scale.

HAD-A =  Hospital anxiety and depression scale- Anxiety.

HAD-D =  Hospital anxiety and depression scale- Depression.

SWD =  Shift work disorder.

According to the crude regression analyses (Tables 3–8), there was a significant and dose-dependent association between working quick returns and insomnia, excessive sleepiness, excessive fatigue and SWD. Excessive fatigue was only significantly associated with working quick returns when the number of quick returns was higher than 30. Anxiety and depression were not associated with quick returns. Furthermore, the crude analyses showed a relation between having more than 90% work position and insomnia, and that working night shifts for 5 years or less was associated with anxiety. A high number of night shifts was related to SWD. Gender was only associated with SWD, and it was found that men were affected to a greater extent than women. Age was positively associated with depression and SWD, while age was inversely associated with excessive sleepiness. The adjusted logistic regression analyses gave quite similar results (Tables 3–8), but in these analyses excessive fatigue was no longer associated with quick returns. Furthermore, SWD was no longer associated with gender, and depression not significantly related to age. The additional adjusted analyses with quick returns as a continuous variable yielded the same significant findings. There was a weak and negative correlation between the number of quick returns and the number of night shifts during the past year (r = −.10, p<.05).

**Table 3:** Crude and adjusted logistic regression analyses with insomnia as the dependent variable.

		Crude	Adjusted^a^ (N = 1579)
		OR (95% CI)	OR (95% CI)
Age		1.00 (0.99–1.01)	1.00 (0.98–1.01)
Gender	male	1.00	1.00
	female	0.89 (0.66–1.20)	0.87 (0.62–1.22)
Number of night shifts past year		1.00 (1.00–1.00)	1.00 (1.00–1.00)
Years with night work	<5	1.00	1.00
	>5	0.95 (0.78–1.16)	0.99 (0.80–1.23)
Fraction of full position	50–75%	1.00	1.00
	76–90%	1.23 (0.92–1.64)	1.34 (0.96–1.86)
	>90%	**1.45 (1.18–1.77)**	**1.36 (1.07–1.72)**
Number of quick returns past year	0	1.00	1.00
	1–30	**1.41 (1.06–1.87)**	**1.44 (1.06–1.96)**
	>30	**1.67 (1.27–2.21)**	**1.57 (1.16–2.12)**

**Table 4:** Crude and adjusted logistic regression analyses with excessive sleepiness as the dependent variable.

		Crude	Adjusted^a^ (N = 1535)
		OR (95% CI	OR (95% CI)
Age		**0.99 (0.97–0.99)**	**0.99 (0.97–1.00)**
Gender male	male	1.00	1.00
	female	1.43 (0.99–2.05)	1.47 (0.99–2.17)
Number of night shifts past year		1.00 (1.00–1.00)	1.00 (1.00–1.01)
Years with night work	<5	1.00	1.00
	>5	0.89 (1.71–1.11)	0.93 (0.73–1.18)
Fraction of full position	50–75%	1.00	1.00
	76–90%	1.08 (0.85–1.65)	1.18 (0.81–1.71)
	>90%	1.21 (0.96–1.53)	1.28 (0.97–1.67)
Number of quick returns past year	0	1.00	1.00
	1–30	**1.68 (1.18–2.40)**	**1.53 (1.05–2.23)**
	>30	**1.93 (1.36–2.72)**	**1.78 (1.24–2.57)**

**Table 5:** Crude and adjusted logistic regression analyses with excessive fatigue as the dependent variable.

		Crude	Adjusted^a^ (N = 1560)
		OR (95% CI)	OR (95% CI)
Age		0.99 (0.98–1.00)	0.99 (0.98–1.00)
Gender	male	1.00	1.00
	female	1.25 (0.91–1.71)	1.11 (0.78–1.57)
Number of night shifts past year		1.00 (1.00–1.00)	1.00 (1.00–1.00)
Years with night work	<5	1.00	1.00
	>5	0.85 (0.70–1.05)	0,94 (0.75–1.17)
Fraction of full position	50–75%	1.00	1.00
	76–90%	1.20 (0.88–1.61)	1.21 (0.87–1.70)
	>90%	1.13 (0.92–1.39)	1.05 (0.83–1.34)
Number of quick returns past year	0	1.00	1.00
	1–30	1.07 (0.80–1.45)	1.03 (0.75–1.42)
	>30	**1.39 (1.05–1.86)**	1.30 (0.95–1.76)

**Table 6:** Crude and adjusted logistic regression analyses with anxiety as the dependent variable.

		Crude	Adjusted^a^ (N = 1566)
		OR (95% CI)	OR (95% CI)
Age		0.99 (0.98–1.01)	1.00 (0.98–1.01)
Gender	male	1.00	1.00
	female	1.44 (0.95–2.20)	1.28 (0.82–2.01)
Number of night shifts past year		1.00 (0.99–1.00)	1.00 (0.99–1.00)
Years with night work	<5	1.00	1.00
	>5	**0.72 (0.56–0.92)**	**0.75 (0.58–0.98)**
Fraction of full position	50–75%	1.00	1.00
	76–90%	1.18 (0.82–1.70)	1.09 (0.72–1.64)
	>90%	1.11 (0.86–1.44)	1.16 (0.86–1.56)
Number of quick returns past year	0	1.00	1.00
	1–30	0.92 (0.64–1.33)	0.85 (0.58–1.25)
	>30	1.10 (0.77–1.56)	0.94 (0.65–1.37)

**Table 7:** Crude and adjusted logistic regression analyses with depression as the dependent variable.

		Crude	Adjusted^a^ (N = 1568)
		OR (95% CI)	OR (95% CI)
Age		**1.02 (1.00–1.04)**	1.02 (0.99–1.04)
Gender	male	1.00	1.00
	female	0.88 (0.53–1.47)	0.90 (0.52–1.56)
Number of night shifts past year		1.00 (1.00–1.01)	1.01 (1.00–1.01)
Years with night work	<5	1.00	1.00
	>5	1.05 (0.74–1.48)	1.02 (0.69–1.49)
Fraction of full position	50–75%	1.00	1.00
	76–90%	0.87 (0.50–1.52)	0.78 (0.42–1.45)
	>90%	1.14 (0.80–1.64)	1.10 (0.73–1.66)
Number of quick returns past year	0	1.00	1.00
	1–30	1.01 (0.60–1.67)	1.01 (0.59–1.73)
	>30	1.08 (0.66–1.77)	1.05 (0.62–1.76)

**Table 8:** Crude and adjusted logistic regression analyses with shift work disorder as the dependent variable.

		Crude	Adjusted^a^ (N = 1560)
		OR (95% CI)	OR (95% CI)
Age		**1.01 (1.00–1.03)**	**1.02 (1.00–1.03)**
Gender	male	1.00	1.00
	female	**0.69 (0.51–0.94)**	0.83 (0.58–1.17)
Number of night shifts past year		**1.01 (1.01–1.01)**	**1.01 (1.01–1.01)**
Years with night work	<5	1.00	1.00
	>5	**1.27 (1.04–1.57)**	1.17 (0.93–1.47)
Fraction of full position	50–75%	1.00	1.00
	76–90%	0.84 (0.62–1.14)	0.95 (0.68–1.34)
	>90%	0.92 (0.75–1.13)	0.92 (0.72–1.17)
Number of quick returns past year	0	1.00	1.00
	1–30	**1.68 (1.22–2.32)**	**1.88 (1.33–2.67)**
	>30	**2.53 (1.85–3.45)**	**2.86 (2.03–4.03)**

OR =  Odds ratio.

CI =  Confidence interval.

a) All predictor variables (age, gender, number of night shifts past year, years with night work, fraction of full position and number of quick returns past year) were entered together in the adjusted analyses.

## Discussion

The main findings of the present study were the positive associations between quick returns and insomnia, excessive sleepiness, excessive fatigue and shift work disorder. Increased number of quick returns was associated with an increased prevalence of these complaints. There was, on the other hand, no evidence that neither anxiety nor depression were related to quick returns.

### Insomnia

According to our hypothesis, a significant relationship between quick returns and insomnia was shown. There are no previous studies on the relationship between insomnia and quick returns in particular, and the findings in the present study are therefore unique. Several studies have, however, been conducted on the relationship between shift work and insomnia. For instance, a study by Härmä et al. concluded that insomnia is more common among 2- and 3-shifts workers compared to regular day workers [32]. Our study suggests that quick returns may be one important factor explaining why shift work is related to insomnia. Our data did not show a clear association between the number of night shifts the past year and insomnia. This may indicate that night work is less important for insomnia than quick returns.

### Excessive sleepiness

We hypothesized that quick returns would be associated with excessive sleepiness. The logistic regression analyses confirmed this hypothesis. This result has also been suggested in other studies. In a narrative review, Sallinen et al. suggest that quick returns are associated with short sleep and increased sleepiness [10]. This review was based on several observational studies of shift work. Furthermore, numerous studies have concluded that there is a positive relation between sleepiness and shift work in general. For instance, a study by Åkerstedt (1988) concluded that shift work was associated with increased subjective, behavioral, and physiological sleepiness. It was suggested that the effects were due to both circadian disturbance and sleep loss [33]. These studies on sleepiness and shift work did, however, not look at sleepiness and quick returns in particular. Our data suggest that quick returns may be a particularly important contributing factor to excessive sleepiness. Similar to insomnia, number of night shifts the past year was not associated with excessive sleepiness.

### Excessive fatigue

We hypothesized that many shifts with short rest in-between might lead to excessive fatigue. In the present study, there was a positive association between excessive fatigue and working more than 30 quick returns during the past year in the crude analysis. A study by Barton and Folkard is in accordance with the result from the present study as they reported that shift work systems that incorporated quick returns were associated with fatigue [17]. We found, however, no evidence that excessive fatigue was related to working less than 30 quick returns the past year. Thus, a work schedule with a few quick returns may not lead to excessive fatigue.

### Anxiety and depression

Regarding mental health, we hypothesized that a work schedule with many quick returns would result in increased anxiety and depression. This hypothesis was not supported by the data, as there was no significant association between working quick returns and neither anxiety nor depression. No previous studies have been conducted on the relationship between anxiety/depression and quick returns in particular. However, Hakola and colleagues found an increase in mental workability (i.e., enjoying social encounters and having a positive outlook) in relation to a reduction in number of quick returns [13]. A study by Bara and Arber found, however, an nonspecific, gender dependent association between shift work and mental health [11]. The study concluded that the mental health among males working various shifts was better than for the females.

### Shift work disorder

Shift work disorder is a sleep disorder related to the work schedule, and we therefore expected to find a relation between quick returns and shift work disorder. The regression analyses confirmed our hypothesis. The chance of having SWD was almost three times higher in the group of nurses who had worked more than 30 quick returns during the last year compared to the group who never worked quick returns. Shift work disorder occurs when an individual is not able to synchronize their internal clock with the work schedule. Working quick returns may be a challenge in this respect, and we suggest that the relation between quick returns and SWD is due to this. A study by Gumenyuk and colleagues demonstrated how SWD is associated with incomplete adaptation of the circadian rhythm [21]. Because of the restricted opportunity to adjust to different sleep-wake rhythms, quick returns may prevent appropriate circadian adjustment to shift work. In this way, quick returns may, because of their very nature, lead to SWD.

### Quick returns and sleep

Previously, several studies have been focusing on the association between night work and sleep related complaints. The results in the present study did, however, suggest that quick returns were more strongly associated with insomnia, excessive sleepiness and excessive fatigue than working night shifts. This implicates that quick returns may be just as important for sleep related complaints as night work, and further research should therefore aim to focus more on quick returns. Not many studies have compared the effect of quick returns and night work. However, a previous study did show that quick returns were just as strongly associated to SWD as night work [12].

While night work in itself does not necessarily limit the nurses' time to rest, quick returns by definition restrict the nurses' opportunity for sleep and other non-work activity in-between shifts. Sleep loss may be one of the most important factors explaining the association with insomnia, sleepiness, fatigue and shift work disorder. Studies have reported that quick returns shorten sleep [14], [34]. Furthermore, studies have reported that quick returns are associated with disrupted, restless and inadequate sleep [17], [35]. In other words, quick returns are associated with both shorter and more disrupted sleep. Work related stress may prolong sleep onset latency, and thereby reduce the sleep duration. Like shift work in general, quick returns may also lead to disturbances of the circadian rhythm. A mismatch in the circadian rhythm may lead to sleep/wake disturbances and internal desynchronization [36]. As evident from the minor negative correlation between number of quick returns and night shifts, nurses with a high number of quick returns did not necessarily have a high number of night shifts. Nurses rotating between day- and evening shifts had many quick returns per year, but no scheduled night shifts. Meanwhile, those working permanent nights had many night shifts, but no scheduled quick returns. Only the three-shift rotation schedule included both quick returns and night shifts.

Since there was a high number of participants in our study, it is of importance to address the clinical relevance of the findings, which can be evaluated in terms of the magnitude of the odds ratios. The difference between having no quick returns versus having more than 30 per year was substantial. For example having more than 30 quick returns per year constituted a three times higher risk of SWD than having no quick returns.

Despite all this, work schedules featuring quick returns are often popular among the workers, as the compressed working time may give longer periods of rest between the working periods. Still, given the effects on health found in the present study, quick returns should be avoided. This is in accordance with a study by Hakola et al. reporting that reduction of the number of quick returns had a positive effect on the physical, mental and social well-being of nurses [13]. Quick returns should, in other words, not be incorporated in the nurses work schedule, and the minimum time in-between shifts should be 11 hours. Further research should aim to assess if workplace strategies to reduce the number of quick returns will reduce complaints of insomnia, sleepiness, fatigue and shift work disorder.

### Strength and limitations

So far, the present study is one of the largest studies investigating the effects of quick returns on sleep and mental health. We used well-validated and standardized instruments, and we also had a large, homogenous sample of nurses. Because of the homogenous sample, the influence of confounding variables, such as work schedule, workload and environment, was limited. This homogeneity does, however, make generalization to other occupations more problematic. Generalization to the male population is also problematic, as only 10% of the participants in the present study were men.

To strengthen the validity of our conclusion, we included number of night shifts during the past year, years with night work experience, and work fraction as predictor variables in the adjusted logistic regression analyses. In this way we controlled for these variables when investigating the associations between quick returns and insomnia, excessive sleepiness, excessive fatigue and shift work disorder. We also adjusted for gender, as men and women tend to answer questionnaires differently under similar work conditions. Furthermore, we adjusted for age, but it should be noted that the mean age of the participants in the present study was low, only 33.1 years. This may have affected the results since older and more experienced nurses may have developed better coping strategies to handle shift work and quick returns.

The design of the present study was cross-sectional, and it is therefore not possible to make conclusions about causal directions. A healthy worker effect is likely to be present in our study as nurses who develop severe problems related to shift work, may change to other work places and/or work schedules. Despite this possible healthy worker effect, we found clear associations between quick returns and health problems.

The response rate in the present study was low, only 38.1%. According to a review on response rates, there is a need of further investigation if the response rate is 60%+/−20% [37]. Although 38.1% is not far below the norm given in this review, the low response rate must still be taken into account. The low response rate might have caused a selected population. There are, however, indications that the sample is representative of Norwegian nurses. The sample shares important characteristics (i.e. distribution of gender and mean weekly work hours) found in the Norwegian nurse population as a whole [38]. We cannot exclude the possibility that the nurses who participated had more problems with sleep and mental health than the average, but studies show that non-participation in studies like this is normally associated with a poorer health status [39]. It might also be possible that nurses with anxiety and depression did not answer the study. However, in the present study, the mean total scores on the HAD-A and HAD-D were 4.7 and 2.8, respectively. The anxiety score was slightly above, and the depression score slightly below the mean score for the general Norwegian population [40]. These minor differences between the general population and our sample make the assumption of few respondents with anxiety and depression less likely.

All the results in the present study were based on data collected with the same questionnaires, and we can therefore not exclude the possibility of common method bias. This bias refers to the fact that variance observed may be due to the method of measurement rather than to the constructs the method represents [41]. Furthermore, all information in the present study was self-reported. Self-reports always entail a risk of recall bias. Some nurses may, in other words, have reported an incorrect number of quick returns. It is, however, likely that the number of quick returns that they reported was a good estimate of the correct number. Symptoms indicating insomnia, excessive sleepiness, excessive fatigue, anxiety, depression and shift work disorder were also self-reported. Since there were no clinical interviews, sleep diaries etc., we could not formally diagnose any of the participants. The questionnaires do however give a good indication of the diagnosis. The scales measuring insomnia, sleepiness, fatigue, anxiety and depression are all well-established tools used in many epidemiological studies. SWD was investigated by using three questions adhering to the core criteria of SWD. This method has been found to be sufficient when assessing SWD caseness in epidemiological studies [12], [30].

In conclusion, we found significant associations between quick returns and insomnia, excessive sleepiness, excessive fatigue and shift work disorder. Increased number of quick returns was associated with an increased number of these complaints. Similar findings were seen even when we controlled for the effects of age, gender, number of night shifts past year, years of night work experience, and fraction of full position. Further research should aim to experimentally investigate the effect of quick returns on sleep and other health outcomes by holding all other shift work related variables constant. Also, effects of workplace strategies to reduce the number of quick returns should be investigated in future studies.
